# Seasonal Patterns of Zoonotic Cutaneous Leishmaniasis Caused by *L*. *major* and Transmitted by *Phlebotomus papatasi* in the North Africa Region, a Systematic Review and a Meta-Analysis

**DOI:** 10.3390/microorganisms10122391

**Published:** 2022-12-02

**Authors:** Ahmed Karmaoui, Denis Sereno, Samir El Jaafari, Lhoussain Hajji

**Affiliations:** 1 Bioactives, Health and Environmental, Epigenetics Team, Faculty of Sciences and Techniques, Errachidia (UMI) and Moroccan Center for Culture and Sciences, Moulay Ismail University, Meknes 50050, Morocco; 2 InterTryp, IRD-CIRAD, Parasitology Infectiology and Public Health Research Group, University Montpellier, 34000 Montpellier, France; 3 Cluster of Competency on Health and Environment, Moulay Ismail University, Meknes 50050, Morocco; 4 Bioactives, Health and Environmental Laboratory, Epigenetics Team, Moulay Ismail University, Meknes 50050, Morocco

**Keywords:** zoonotic cutaneous leishmaniasis, *Phlebotomus papatasi*, seasonal transmission, *L. major*, Africa region, meta-analysis

## Abstract

Background: In North African countries, zoonotic cutaneous leishmaniasis (ZCL) is a seasonal disease linked to *Phlebotomus papatasi*, Scopoli, 1786, the primary proven vector of *L. major* dynamics. Even if the disease is of public health importance, studies of *P. papatasi* seasonal dynamics are often local and dispersed in space and time. Therefore, a detailed picture of the biology and behavior of the vector linked with climatic factors and the framework of ZCL outbreaks is still lacking at the North African countries’ level. Our study aims to fill this gap via a systematic review and meta-analysis of the seasonal incidence of ZCL and the activity of *P. papatasi* in North African countries. We address the relationship between the seasonal number of declared ZCL cases, the seasonal dynamic of *P. papatasi*, and climatic variables at the North African region scale. Methods: We selected 585 publications, dissertations, and archives data published from 1990 to July 2022. The monthly incidence data of ZCL were extracted from 15 documents and those on the seasonal dynamic of *P. papatasi* from 11 publications from four North African countries. Results: Our analysis disclosed that for most studied sites, the highest ZCL incidence is recorded from October to February (the hibernal season of the vector), while the *P. papatasi* density peaks primarily during the hot season of June to September. Overall, at the North African region scale, two to four months laps are present before the apparition of the scars reminiscent of infection by *L. major*. Conclusions: Such analysis is of interest to regional decision-makers for planning control of ZCL in North African countries. They can also be a rationale on which future field studies combining ZCL disease incidence, vector activity, and climatic data can be built.

## 1. Introduction

Cutaneous leishmaniasis (CL), a skin infection provoking ulcers on exposed body parts, affects annually between 600,000 to 1 million new cases on a global scale [1]. In North African countries, Morocco, Algeria, Tunisia, and Libya, the disease is caused by *L. major*, *L. tropica*, and *L. infantum*, which belong to the Leishmania genus [2]. Cutaneous leishmaniasis caused by *L. major* remains the most frequent in North Africa and Middle East countries. Still, they present a high incidence rate in other territories, such as Afghanistan [3], Iran [4], and Saudi Arabia [5]. One or more lesions appear after the bite by an infected sand fly, and the inflammation causes scarring if not treated, which leads to traumatic psychological impacts. Shaw’s jird (*Meriones shawi*) and the fat sand rat (*Psammomys obesus*) are the identified reservoirs, while *P. papatasi* is the proven vector of *L. major* [6,7]. This disease affects regions with semi-arid and arid climates in North African countries, especially in the poorest provinces. It is estimated that 97.8% of cases concentrate in areas with poor socio-economic conditions, below 725 m, and arid semi-arid climates [8]. The infection affects, more commonly, younger humans and males. Most ZC cases are aged < 10 years, males, with ulcers primarily present on the face and hands [4]. ZCL is endemic in rural areas, with lesions and scars appearing between August and January [9] in arid regions [10]. Socioeconomic conditions [11], anthropogenic disturbance in the peri-urban area [12], and topography [13] are also associated with the disease incidence. Vegetation, wind speed, and altitude are significant factors in forecasting ZCL cases [14].

In North African countries, zoonotic cutaneous leishmaniasis caused by *L. major* remains among the most widespread neglected tropical diseases impacting public health. The geographical dispersion of the disease is primarily affected by ecological and socioeconomic drivers that favor vector proliferation and host-reservoir sheltering conditions. In addition, seasonal climatic conditions influence ZCL incidence and dispersion [15] via the ecological conditions required for the vector and reservoirs. These factors were addressed in Morocco, Algeria [10], and Tunisia [16]. The world health organization (http://www.emro.who.int/neglected-tropical-diseases/information-resources-leishmaniasis/cl-factsheet.html; accessed on 14 July 2022) reported the incubation period of *L. major* needs at least one week and usually less than four months, while *L. tropica* requires at least one week and usually 2–8 months [17].

Most published research papers focus on localized geographical areas in a restricted time range. Here, we collected data on the proven vector of ZCL, *P. papatasi*, whose seasonal activity causes seasonal outbreaks at the North African region scale. To our knowledge, no studies dealing with these aspects were undertaken on such a broad geographic scale. We further analyzed the interplay between seasonal ZCL cases number, *P. papatasi* density, and climatic data.

## 2. Materials and Methods

### 2.1. Systematic Review, Data Set Collection, and Localization of Sites

#### 2.1.1. Systematic Review

**Figure 1:**
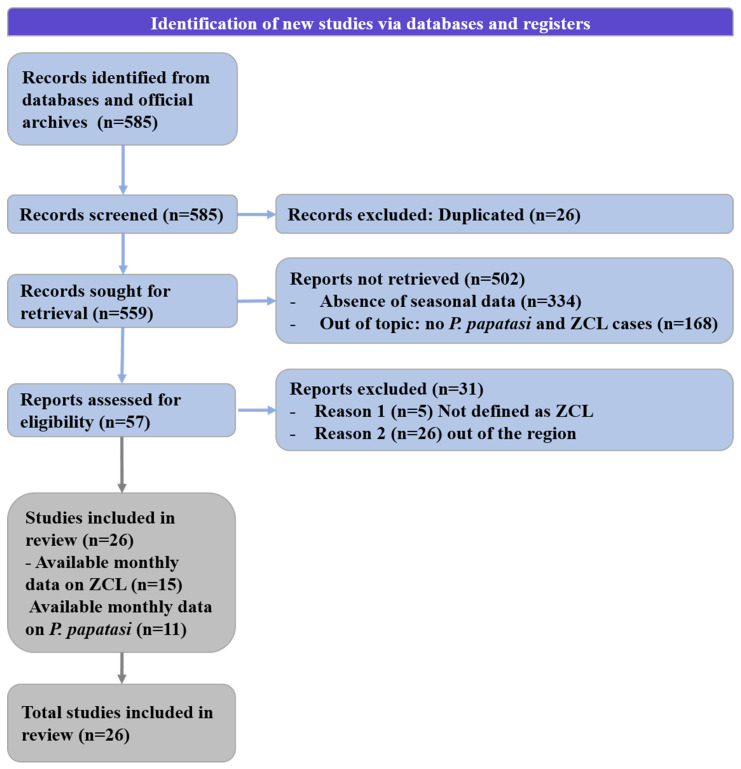
Flow chart of the current study following the recent updates of the PRISMA guidelines of 2020.

The current study was based on a systematic review following the guidelines of the PRISMA initiative (2020) [18] (Figure 1) and meta-analyses [19].

Monthly data on ZCL case number (Table 1) and *P. papatasi* density (Table 2) were extracted from selected publications. The keywords used in this study are “leishmaniasis”, “ZCL seasonal transmission”, “cutaneous leishmaniasis”, “*L. major*”, “sand fly”, “vectors of Leishmaniasis”, ”*P. papatasi*” combined with “North Africa”, and the countries of the area, including from north to east “Morocco”, ”Algeria”, “Tunisia”, and “Libya”. The searches were performed on 11–14 July 2022 using PubMed, Web of Science, Scopus, and Google Scholar. Only studies with available monthly data were included from an initial panel of 585 scientific publications published between 1990 and July 2022. From this panel, 15 documents gathered data on the monthly incidence of ZCL, while only 11 presented data on the seasonal dynamic of *P. papatasi* in African countries. The annual distribution of ZCL cases in the considered area was compiled from 1995 to 2020 (the available data).

#### 2.1.2. Data Set Collection and Sites Localization

**Figure 2:**
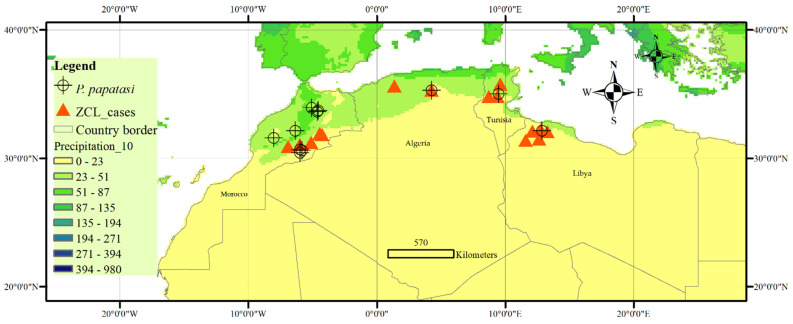
Localization of the North African countries with seasonal activity data of ZCL cases and *P. papatasi* with the annual distribution of precipitation (mm). ZCL seasonal data: the current study. Climatic data source: ESRI grids, resolution 10 min according to WorldClim 1.4 (current conditions) http://worldclim.com/current, accessed on 15 October 2022 [41] is licensed under a Creative Commons Attribution-ShareAlike 4.0 International License.

Quantitative and qualitative information from the selected publications were extracted and presented in two tables; a map of the extracted data is shown in Figure 2.

The geographic information, including altitude, latitude, and longitude, was extracted from the literature (Table 1 and Table 2, and Figure 1), and climatic variables (monthly maximum temperature, minimum temperature, precipitation, and relative humidity) in the relevant areas were collected from the Tutiempo Network, S.L.

Data were processed using the Arc-GIS software (Figure 2, Table 1 and Table 2) and combined with the regional annual precipitation (Figure 2). Most sites with available published data are localized in semi-arid to arid areas where the rainfall is low and irregular in time and space, and the temperature ranges from moderate to high.

### 2.2. Normalization of ZCL Case Number and/Vector Density (Nj)

Data on monthly ZCL cases number and *P. papatasi* density were extracted from tables or digitized using ‘digitizelt’ software and represented by country. However, the original values of these different sources are heterogeneous, which requires the normalization of data to be comparable. Therefore, equation 1 was used to calculate the normalized incidence value of ZCL standardizing extracted values for monthly ZCL cases and *P. papatasi* activity on a scale ranging from 0 to 1, where 0 refers to the absence of data or no recorded activity, and 1 indicates the highest activity. For example, the normalized values of a month’s incidence and vector activity are a ratio between the extracted values for that month and the month with the maximal value.

Nj = [ZCL cases or vector activity in a month]/[Month with maximal ZCL cases or vector activity](1)

### 2.3. Statistical Analysis

The Pearson correlation coefficient and R2, the coefficient of determination, were analyzed with the social science statistics calculator, R.

## 3. Results and Discussion

### 3.1. Annual Distribution of ZCL Cases in the North African Region

**Figure 3:**
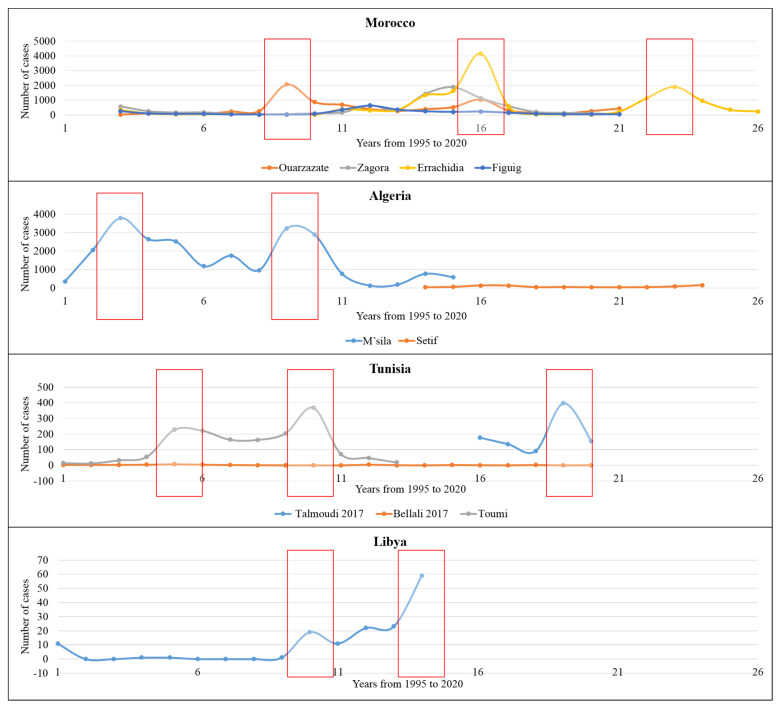
The evolution of ZCL cases in North African countries from 1995 to 2020 (26 years) as revealed by the systematic review and meta-analysis. The red box refers to the year with the peak of incidence.

We compiled annual data on published ZCL case numbers in North African countries for 26 years (from 1995 to 2020) (Figure 3). In addition, data from four oasis sites (Ouarzazate, Zagora, Errachidia, and Figuig) were considered for Morocco. As a result, three principal peaks were recorded in 2003, 2010, and 2017. In Algeria, data from two sites point to two prominent peaks in 1997 and 2003, while data from Tunisian sites depict three primary peaks in 1999, 2004, and 2013. Finally, data published from Libya shows two major peaks in 2004 and 2008 (Figure 3).

An overall decreasing trend in ZCL incidence was observed during the period studied and in the sites we reviewed (Supplementary Figure S1). However, if the average ZCL case number followed a decreasing trend in Algeria and Tunisia, the opposite is reported in Morocco and Libya; overall, the decreasing trend was recorded in North Africa, which can be related to interventions aimed at combating the disease.

### 3.2. Monthly Distribution of P. papatasi in the North African Region

**Figure 4:**
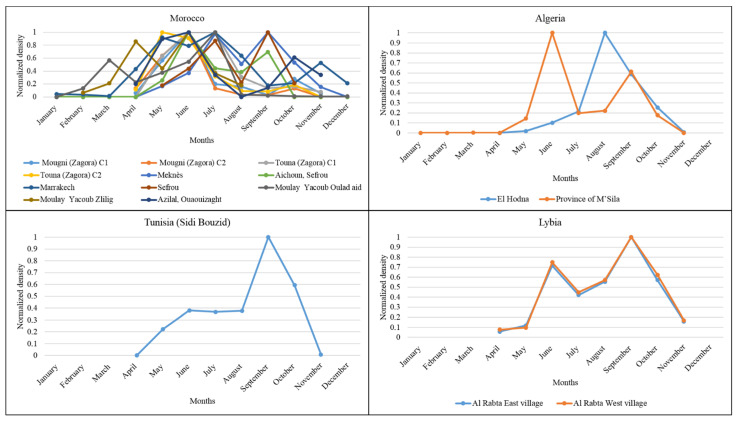
The normalized monthly density of the *P. papatasi* with 0 indicates no data/absence of activity, and 1 shows high density in the studied sites in the Northern African countries.

Data collected on the seasonal *P. papatasi* dynamic depicted bi- to tri-modal activity from March to November (Figure 4). They point to a higher *P. papatasi* density during the hot period in the Moroccan sites. In the Algerian sites studied, El Honda recorded a monomodal activity, with the maximum activity in August. In M’Sila, a bi-modal distribution with a maximal peak in June is recorded. Sidi Bouzid, Tunisia, displays seasonal activity with a density peak in September. The two sites studied in Libya display a bi-modal distribution with maximal density in June and September. These results show *P. papatasi* density peaks primarily during the hot season in the North African region.

Our analysis at the North African geographic scale depicts a seasonal activity of *P. papatasi* with maximal densities during the hot season. In North African countries, high densities are recorded in October and November in Marrakech, Morocco [34], and in August and September in Tunisia and Algeria [27,28]. While in Egypt, density peaks are recorded in July [42], while in Saudi Arabia and Iran, they occur in May and August [43].

### 3.3. Monthly Records of ZCL Cases in the North African Region

**Figure 5:**
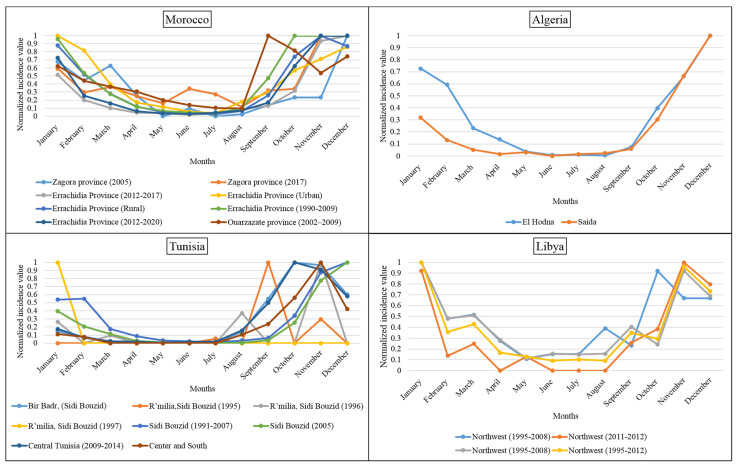
Seasonal distribution of ZCL cases, total values of all sites of the North African region.

Morocco and Tunisia are the North African countries with the highest published data, followed by Libya and Algeria. In the Moroccan sites, the high incidence of ZCL was recorded from October to February, coinciding with the vector’s hibernal season. This pattern was also found in the Algerian sites. However, Libyan and Tunisian sites display a pattern with a high incidence ranging from October to January (Figure 5). For the whole region, ZCL recorded a high incidence between October and January (Figure 5). This indicates a contrast between the maximal activity of the vector (*P. papatasi*) and the apparition of the ZCL lesions.

This increase in seasonal incidence in the North African countries was also found in other regions. For example, in Afghanistan (Mazar-e Sharif), most cases of ZCL occur in mid-October [3], while in Iran (Golestan Province), the high incidence is recorded in September and October [44]. In addition, hot temperatures occurring after a wet season could increase the number of ZCL cases [15].

**Figure 6:**
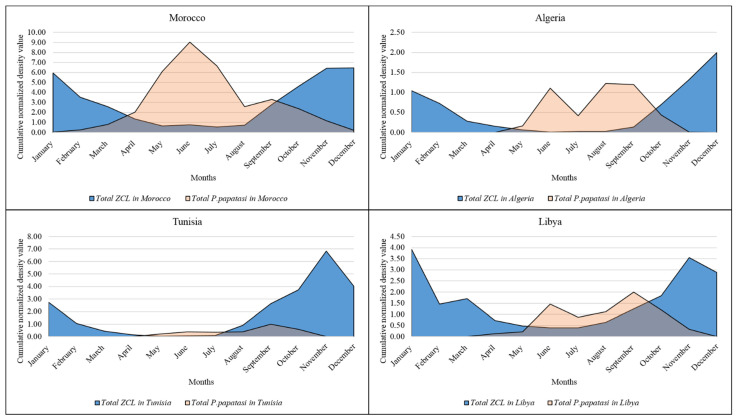
Seasonality of ZCL cases and *P. papatasi* sand fly vector density in the studied countries. Data were expressed as a cumulative normalized density value of stations under study.

To highlight differences between the monthly activity of the vector (*P. papatasi*) and the number of ZCL cases, a combination of the normalized average values per country and within the North African region was performed (Figure 6). Differences may add information helping to delineate the laps between the vector emergence and biting (the infection by the *L. major*) and the apparition of the lesion(s) following a medical consultation. There are great laps in time between sandfly density and ZCL incidence with differences between region countries. *P. papatasi* displays a bi-modal pattern of activity, with the highest activity in June and September, while maximum ZCL cases are recorded in November and January (Figure 6).

**Figure 7:**
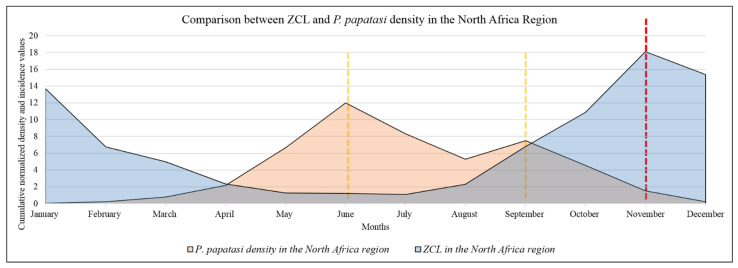
Seasonality in ZCL case number and *P. papatasi* density at the North African scale. Yellow dotted lines refer to the peaks of P. papatasi density and red dotted line indicates the peak of ZCL cases.

Data collected from the North African countries included in this study display a bi-modal distribution of *P. papatasi* in June and September; concomitantly, the peak of ZCL cases was recorded in November (Figure 7).

Overall, in the North African region, two to four months laps are recorded between lesions and scars reminiscent of *L. major* infection appearance and peak density of *P. papatasi*. Such a lap was also recorded in Afghanistan (Mazar-e Sharif) with an incubation period of 8–12 weeks. In Germany, the incubation period for ZCL was reported to be seven weeks [3].

### 3.4. Association of ZCL Incidence and the Climatic Parameters in North Africa

**Figure 8:**
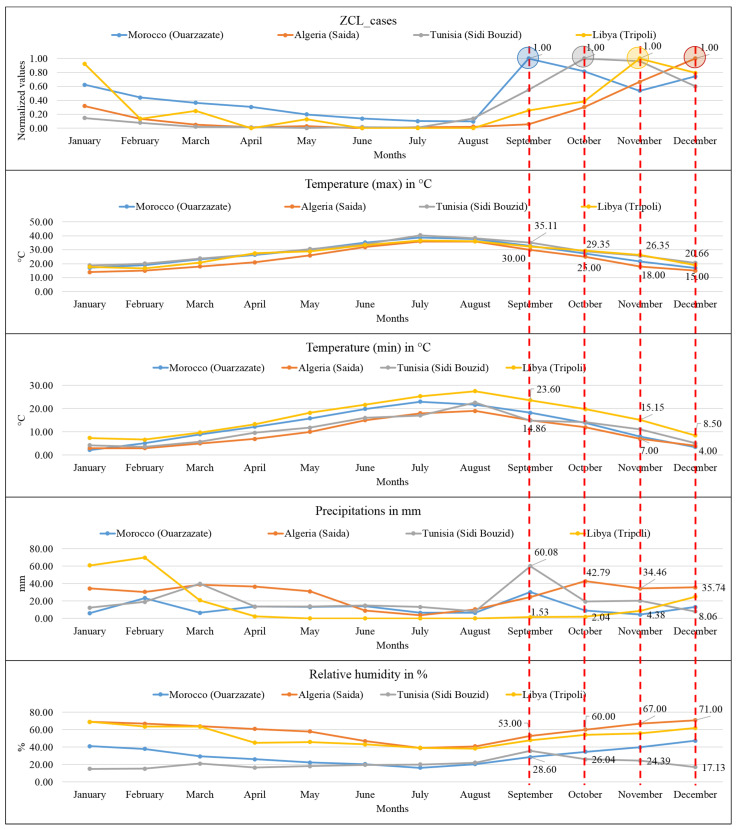
Association between seasonal ZCL incidence and the climatic parameters. Red dotted lines indicate the association between the peaks of ZCL cases and climatic parameters.

The distribution in time and space of the ZCL follows a seasonal dynamic depending on and following climatic variables, such as rainfall, temperature, or relative humidity. Generally, the rise in cases starts in August and reaches a maximum in September in the Moroccan sites, October in Tunisia and Libya, and December in Algeria. In these countries, the maximum number of cases coincides with the highest amount of rainfall (Figure 8).

In the Moroccan site (Ouarzazate), the peak of ZCL cases was recorded in September and coincided with 32.73 °C (maximum temperature), 18.26 °C (minimum temperature), 30.32 mm (monthly precipitation), and 28.6% (relative humidity) (Figure 8). In the Algerian site (Saida), the peak of ZCL cases was recorded in June and coincided with 15 °C (maximum temperature), 4 °C (minimum temperature), 35.74 mm (monthly precipitation), and 71% (relative humidity). In the Sidi Bouzid site (Tunisia), the peak of ZCL cases density was recorded in October and coincided with 28.79 °C (maximum temperature) and 14.24 °C (minimum temperature), 19.47 mm (monthly precipitation), and 26.04% (relative humidity). In the Libyan site (Al Rabta East), the peak of ZCL cases density was recorded in November. It coincided with 26.35 °C (maximum temperature), 15.15 °C (minimum temperature), 8.76 mm (monthly precipitation), and 55.85% (relative humidity) (Figure 8).

The calculated Pearson correlation coefficient displays a moderate to a substantial likelihood of ZCL cases being associated with maximum temperature, precipitation, and relative humidity in Moroccan sites and a positive correlation and mild likelihood with relative humidity in Algerian sites. In contrast, in the Libyan area, a highly positive association is recorded with precipitation (Table 3). In addition, such associations with rainfall and maximum temperatures [45], minimum temperature [46], and aridity [10] have also been disclosed.

### 3.5. Association of the P. papatasi Activity and the Climatic Parameters in the North African Region

**Figure 9:**
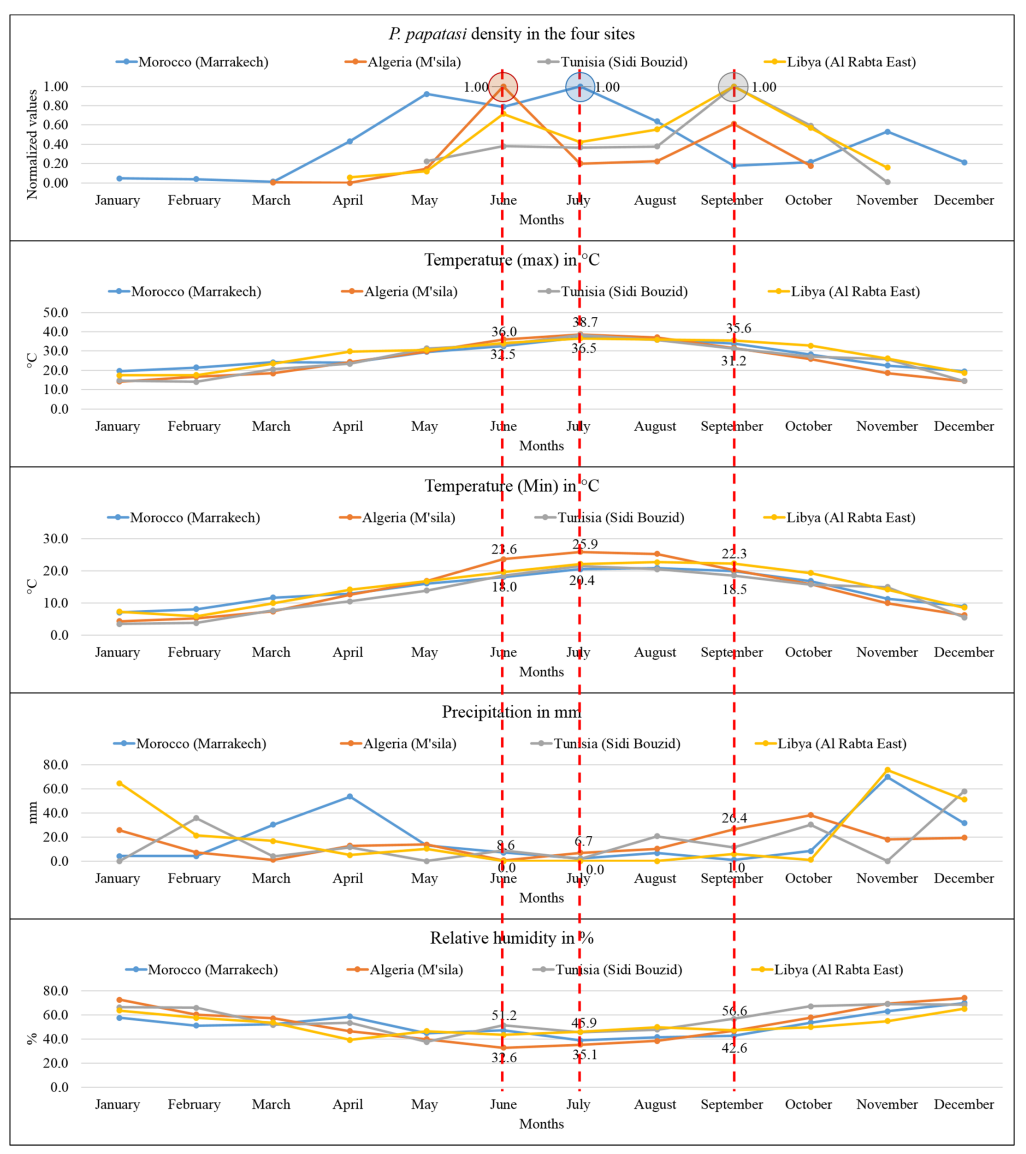
Association between *P. papatasi* activity and climatic variables in the four studied sites, including from top to down; monthly *P. papatasi* activity, monthly maximum and minimum temperatures in °C, monthly precipitation in mm, and monthly relative humidity in %. Red dotted lines indicate the association between the peaks of *P. papatasi* density and climatic parameters.

The distribution in time and space of *P. papatasi* follows a seasonal fluctuation and is dependent on climatic variables, such as rainfall, temperature, and relative humidity. We tested the correlation of these parameters with the *P. papatasi* density using the available monthly data from four North African country sites. In these sites, the vector density peaks from May to September. In the Moroccan site (Marrakech), the rise of *P. papatasi* density was recorded in July and coincided with 37 °C (maximum temperature), 20.4 °C (minimum temperature), 2.2 mm (monthly precipitation), and 38.7% (relative humidity) (Figure 9). In the Algerian site (M’sila), the density peak was recorded in June and coincided with 36 °C (maximum temperature), 23.6 °C (minimum temperature), 0.5 mm (monthly precipitation), and 32.6% (relative humidity). In the site of Sidi Bouzid (Tunisia), the peak was recorded in September and coincided with 31.2 °C (maximum temperature), 18.5 °C (minimum temperature), 11.3 mm (monthly precipitation), and 56.6% (relative humidity). Finally, in the Libyan site (Al Rabta East), the peak of *P. papatasi* density was recorded in September. It coincided with 35.6 °C (maximum temperature), 22.3 °C (minimum temperature), 5.7 mm (monthly precipitation), and 47.3% (relative humidity) (Figure 9).

These findings support that *P. papatasi* activity occurs during the hot season, where both minimum and maximum temperatures are high, and low temperatures during the wet season prevent vector activity. Such observation was already reported [40]. In addition, *P. papatasi* metabolism and the intravectorial development of Leishmania are primarily influenced by temperature [47]. Our study discloses a significant association between *P. papatasi* density and temperature (maximum and minimum). A moderate positive association is ascertained in Tunisia (Sidi Bouzid) and Libya (Al Rabta East), suggesting a tendency of P. papatasi density to be somewhat linked to high, maximum, and minimum temperatures (Table 4).

Furthermore, a medium to strong positive correlation was recorded for the Moroccan site of Marrakech, while a weak to moderate association was found in the Algerian site. Concerning the precipitation variable, a weak correlation was recorded (Table 4). However, only the Moroccan site showed a moderate positive correlation between *P. papatasi* and relative humidity.

Data analysis further supports the hypothesis of an intricate association between climatic factors, vector density, and disease incidence. It reinforces previous reports on the seasonal activity of *P. papatasi* [48,49] and the impact of precipitation and air temperature as significant factors affecting *P. papatasi* distribution [40,50,51]. In addition, moisture, wind [52], aridity, or surface climate variables [10] also influence vector activity and dispersal capability. All these pieces of evidence forecast climate change as a driver for the expansion of cutaneous leishmaniasis since they can favor contacts between the host, the vector, and human populations [53]. Minimum and maximum temperatures are mainly associated with *P. papatasi* activity, which points to the interest of these as climatic indicators to predict ZCL incidence. The outputs may be used to set up models to forecast the periods of high vector density and, consequently, the risk of ZCL. Further intercountry research efforts are required to monitor the ZCL incidence, abundance, and seasonal density of *P. papatasi* and to collect more local climatic variables, including maximum and minimum temperature, relative humidity, and precipitation.

### 3.6. Strengths and Limitations

North Africa belongs to one of the most impacted geographic areas by ZCL. In this paper, for the first time, the seasonal incidence of *L. major* infection (ZCL) and the activity of its primary proven vector (*P. papatasi*) were investigated at the North African geographic scale. Using data from the literature, we explore associations between disease incidence, vector activity, and climatic factors to delineate underlying factors playing a role in the ZCL seasonal dynamic and its spread. Nevertheless, the nature of the data we analyzed, dispersed in terms of time and geographic coverage, as well as the differences in time steps between data, particularly those concerning *P. papatasi* dynamic, limits the strength of our analyses and, therefore, the conclusions raised by the study.

## 4. Conclusions

Our analysis points out that peaks of ZCL cases occur from October to February (the hibernal season of the vector), while the density of *P. papatasi* peaks mainly in the hot seasons in June and September. Therefore, if the presence of *P. papatasi* does not always imply the existence of ZCL cases, it can be an alert or an indicator of a high risk for ZCL transmission. Therefore, the outputs can be used as a basis for future field studies about ZCL disease risk and management at a regional scale and decision-making in control planning in North Africa.

## Figures and Tables

**Table 1:** Geolocalization of sites whose monthly data of ZCL cases in the North African countries are published. The geographic information includes the latitude, longitude, and altitude (m); for the sites without geographic data, the symbol ‘~’ was used, and the approximate values were estimated from Google Earth.

Country	Zone	Latitude	Longitude	Altitude (m)	Annual Rainfall (mm)	Period	References
Morocco	Zagora province (2005)	~30°18′27.60″ N	~5°52′4.55″ W	500	80	2005	[20]
	Zagora province (2017)	~30°40′20.60″ N	~5°7′4″ W	500	80	2017	[20]
	Errachidia Province (2012–2017)	~31°55′5″ N	~4°26′6″ W	~1037	150	2012–2017	[21]
	Errachidia Province (Urban)	~31°55′46.27″ N	4°26′55.83″ W	~1037	134	2004–2013	[22]
	Errachidia Province (Rural)	~31°51′1″ N	~4°16′4″ W	~1011	134	2004–2013	[22]
	Errachidia Province (1990–2009)	~31°55′46″ N	~4°26′55″ W	~1037	134.28	1990–2009	[10]
	Errachidia Province (2012–2020)	~31°55′48″ N	~4°26′58″ W	~1037	134	2012–2020	[23]
	Ouarzazate province (2002–2009)	~30°55′59″ N	~6°55′10″ W	~1148	146.97	2002–2009	[12]
Algeria	El Hodna	35°18′–35°32′N	4°15′–5°06′ E	400–1800	500–600	1995–2000	[24]
	Saida	~34°50′29.47″ N	~0°8′44.18″ E	~802	353.47	1990–2009	[10]
Tunisia	Bir Badr, Hichria, and Zefzef (Sidi Bouzid)	~34°49′45″ N	~9°22′37″ E	407	228	2009–2015	[25]
	R’milia, Sidi Bouzid (1995)	35°46′ N	9°36′ E	280	260	1995	[26]
	R’milia, Sidi Bouzid (1996)	35°46′ N	9°36′ E	280	260	1996	[26]
	R’milia, Sidi Bouzid (1997)	35°46′ N	9°36′ E	280	260	1997	[26]
	Sidi Bouzid (1991–2007)	~35°02′00″ N	~9°30′00″ E	~373	-	1991–2007	[16]
	Village of Felta, Sidi Bouzid (2005)	~34°49′27″ N	8°42′09″ E	~768	-	2004–2005	[27]
	Central Tunisia (2009–2014)	~34°49′45″ N	~9°22′37″ E	~407	~228	2009–2014	[9]
	Center and South	Large distribution in the center and south of the country				2007–2011	[28]
Libya	Northwest (1995–2008)	Large distribution in the north-western districts of the country.				1995–2008	[29]
	Northwest (2011–2012)	Large distribution in the north-western districts of the country, including Jafara, Tripoli, Misrata, and Nalut.				2011–2012	[30]
	Northwest (1995–2008)	Large distribution in the north-western districts of the country, including Jafara, Tripoli, Misrata, and Nalut.				1995–2008	[30]
	Northwest (1995–2012)	Large distribution in the north-western districts of the country, including Jafara, Tripoli, Misrata, and Nalut.				1995–2012	[30]

**Table 2:** Geolocalization of sites whose monthly density data of *P. papatasi* in the North African countries was extracted. The geographic information includes the latitude, longitude, and altitude (m); for the sites without geographic data, the symbol ‘~’ was used, and the approximate values were estimated from Google Earth.

Country	Zone	Latitude	Longitude	Altitude (m)	Annual Rainfall (mm)	Period	References
Morocco	Ksar Mougni, Tinzouline (Zagora) C1	30°27′3″ N	5°58′26″ W	775	37	2019	[31]
	Ksar Mougni, Tinzouline (Zagora) C2	30°27′3″ N	5°58′26″ W	775	37	2019	[31]
	Touna, Tinzouline (Zagora) C1	30°37′28.2″ N	5°49′56.1″ W	910	26	2019	[31]
	Touna, Tinzouline (Zagora) C2	30°37′28.2″ N	5°49′56.1″ W	910	26	2019	[31]
	Meknes (prefecture)	~33°45′02″ N	~4°34′00″ O	500	660	2016–2017	[32]
	Aichoun, Tazouta, Sefrou	~33°45′22″ N	~5°32′26″ O	750	400	2013–2014	[33]
	Marrakech Urban	31°36 N	8°02 W	471		2002–2003	[34]
	Sefrou	33°39 N	04°38 W	809		2012	[35]
	Moulay Yacoub Oulad aid	~34°05′N	~4°45′ W	345		2011–2012	[36]
	Moulay Yacoub Zlilig	33°57 N	5°05 W	500		2011–2012	[36]
	Azilal province, Ouaouizaght district	32°09′27.26″ N	6° 20′57.58″ O	~900–1200		2010	[37]
Algeria	El Hodna	35°18′−35°32′ N	4°15′– 5°06′	500–800		2004	[38]
	Province of M’Sila	35°18′ and 35°32′ N	4°15′ and 5°06′E			2003/2004	[39]
Tunisia	SidiBouzid	~35°02′01″ N	~9°28′54″ E	350		2005	[27]
Lybia	Al Rabta East village	32°9′46.59″ N	12°50′50.65″ E	300		2012–2013	[40]
	Al Rabta West village	32°9′46.59″ N	12°50′50.65″ E	300		2012–2013	[40]

**Table 3:** Correlation between ZCL cases and climatic parameters in the studied countries.

	Correlation Method	Temperature (Maximum)	Temperature (Minimum)	Precipitation	Relative Humidity
Tunisia (Sidi Bouzid)	R	0.4326	0.3309	0.189	0.3225
	R2	0.1871	0.1095	0.0357	0.104
Algeria (Saida)	R	0.3711	0.2273	0.4185	0.5764
	R2	0.1377	0.0517	0.1751	0.3322
Morocco (Ouarzazate)	R	0.5197	0.2989	0.6254	0.6715
	R2	0.2701	0.0893	0.3911	0.4509
Libya (Tripoli)	R	0.3438	0.2427	0.8641	0.3003
	R2	0.1182	0.0589	0.7467	0.0902

R, Pearson correlation coefficient; and R2 is the coefficient of determination.

**Table 4:** Results of the correlation between *P. papatasi* and climatic parameters as computed with the social science statistics calculator.

	Correlation Method	Temperature (Maximum)	Temperature (Minimum)	Precipitation	Relative Humidity
Tunisia (Sidi Bouzid)	R	0.5119	0.5685	0.498	0.4616
	R2	0.262	0.3232	0.248	0.2131
Algeria (M’SILA)	R	0.494	0.5537	0.4893	0.3296
	R2	0.244	0.3066	0.2394	0.1086
Morocco (Marrakech)	R	0.7509	0.5061	0.3147	0.5725
	R2	0.5639	0.2561	0.099	0.3278
Libya (Al Rabta East)	R	0.5405	0.5309	−0.2077	0.4894
	R2	0.2921	0.2819	0.0431	0.2395

R, Pearson correlation coefficient; and R2 coefficient of determination.

## Data Availability

Not applicable.

## Supplementary Materials

The following supporting information can be downloaded at: https://www.mdpi.com/article/10.3390/microorganisms10122391/s1, Figure S1. The average evolution of ZCL cases in whole North African region from 1995 to 2020 (26 years).
