# Supplementary figures and images for: Seasonal Patterns of Zoonotic Cutaneous Leishmaniasis Caused by L. major and Transmitted by Phlebotomus papatasi in the North Africa Region, a Systematic Review and a Meta-Analysis
Source: Microorganisms. 2022 Dec 2;10(12):2391. doi: 10.3390/microorganisms10122391 (PMC9782821; doi:10.3390/microorganisms10122391)

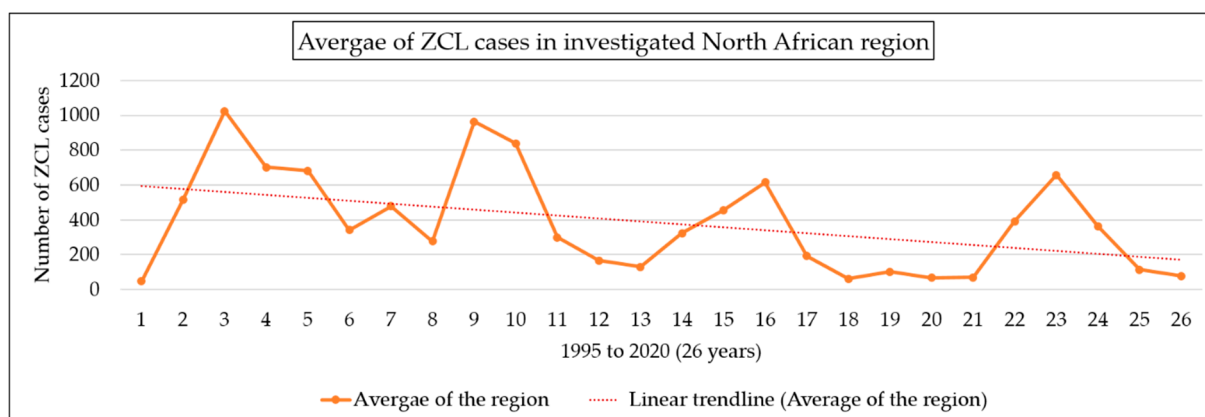

Figure S1. The average evolution of ZCL cases in whole North African region from 1995 to 2020 (26 years),

Supplement: Supplementary file 1 [file microorganisms-10-02391-s001.zip › microorganisms-2076421-supplementary.pdf]
